# The endotoxin/toll-like receptor-4 axis mediates gut microvascular dysfunction associated with post-prandial lipidemia

**DOI:** 10.1186/1472-6793-13-12

**Published:** 2013-11-12

**Authors:** Ping Yi, Jia Pang, Jonathan Steven Alexander, Chantal Rivera

**Affiliations:** 1 Molecular & Cellular Physiology, LSU Health, 1501 King Highway, Shreveport, LA, USA; 2 Microbiology and Immunology, LSU Health, 1501 King Highway, Shreveport, LA 71130, USA; 3 Clinical Integrative and Molecular Gastroenterology (CIMG) Study Section; Digestive, Kidney and Urological Systems Integrated Review Group, Center for Scientific Review, National Institutes of Health, 6701 Rockledge Drive, MSC 7818, Room 2186, Bethesda, MD, USA

**Keywords:** Microcirculation, Post-prandial lipidemia, TLR4, SIRT1

## Abstract

**Background:**

Postprandial lipidemia is important in the development of coronary artery disease (CAD). Consumption of a meal high in monounsaturated fat was correlated with acute impairment of endothelial function. However, the mechanisms underlying impaired endothelial function in the postprandial state have not yet been elucidated. The effects of polyunsaturated fat (corn oil) and monounsaturated fat (olive oil) on vascular dysfunction in intestinal postcapillary venules and arterioles were examined in wild-type (WT) mice, mice genetically deficient in TLR4 (TLR4^-/-^) and mice pre-treated with antibiotics by intravital microscopy which was performed 1.0, 1.5, 2.0, 2.5 hours after oil administration. After intravital microscopy, samples of jejunum were therefore collected to test TLR4, pNF-kB p65 and SIRT1 protein expression by western blotting.

**Results:**

Our findings showed that feeding mono-unsaturated olive oil or polyunsaturated corn oil promoted leukocyte and platelet trafficking in the gut microvasculature, and impaired endothelium-dependent arteriolar vasodilator responses during postprandial lipidemia. The expression of TLR4, pNF-kB p65 was significantly increased in mice gavaged with olive oil at 2 h and was significantly reduced in mice gavaged for 7 days with antibiotics and in TLR4 knockout (TLR4^-/-^) mice. At the same time, SIRT1 protein expression is diminished by feeding olive oil for 2 h, a phenomenon that is attenuated in mice pre-treated with antibiotics and in TLR4^-/-^ mice. Corn oil treated mice exhibited a pattern of response similar to olive oil.

**Conclusions:**

Dietary oils may be negative regulators of SIRT1 which activate the innate immune response through the endotoxin/TLR4 axis. Our findings establish a link between innate immunity (i.e. the endotoxin/TLR4 axis) and epigenetic controls mediated by SIRT1 in the genesis of diet associated vascular stress.

## Background

Along with rates of obesity, the incidence of co-morbid conditions such as cardiovascular disease has also increased annually for more than a decade. Impaired endothelial function is central to atherosclerotic disease processes, and serves as a significant, independent risk factor of future cardiovascular disease and mortality [1,2]. The ingestion of a HFM acutely changes the blood lipid profile and reduces endothelial function for many hours following a meal [3]. Typical eating patterns among obese populations in the US consist of foods that are high in saturated fat, sugars and cholesterol, the so-called “western diet” and the content of saturated fat correlates positively with the incidence cardiovascular disease [4-8]. Postprandial lipidemia is important in the development of coronary artery disease (CAD) because of the known contributions of elevated triacylglycerol-rich plasma lipoproteins and suppressed high density lipoprotein (HDL)-cholesterol concentrations to CAD [9]. Consumption of a meal high in monounsaturated fat was correlated with acute impairment of endothelial function when compared with a carbohydrate-rich meal [10]. The mechanisms underlying impaired endothelial function in the postprandial state are likely multi-factorial [11-14] but are likely to include decreased NO bioavailability as well as increased expression of pro-inflammatory cytokines and endothelial cell adhesion molecules [15].

It is well-known that lifestyle changes that diminish calorie load, such as reduced caloric intake or increased energy expenditure via exercise, are effective means of diminishing cardiovascular disease risk in obese patients. These behavioral changes also positively influence the activity of SIRT1, a class III protein deacetylase. Moreover, and the SIRT1 activator resveratrol has been shown to mediate reduced atherosclerosis [16]. Cell culture and animal studies have demonstrated that SIRT1 exerts protective effects against endothelial dysfunction by preventing stress-induced senescence [17] and increasing eNOS mediated NO production [18]. Moreover, increased expression of SIRT1 blunts high fat diet-induced attenuation of endothelium-dependent relaxation in isolated aortic rings from apoE knockout mice [19]. Taken together, these findings suggest that SIRT1 is cardio-protective; however the molecular mechanisms underlying these beneficial effects have not yet been elucidated.

The TLR family of pattern recognition receptors is critical to host defense against invading pathogens. Ligand interactions with the TLR4 complex result in the recruitment of multiple adaptor molecules to the cell membrane that propagate signaling cascades which result in the translocation of NF-kB to the nucleus where it triggers the synthesis of several inflammatory mediators. Since endotoxin (produced by gut microflora) is a ligand for TLR4, we further hypothesize that vascular defects due to lipidemia might require TLR4. Our hypothesis is that dietary oils negatively regulate SIRT1 by activating an innate immune response via the endotoxin/TLR4 axis.

The present study was therefore designed to identify molecular mechanisms underlying lipid-induced vascular dysfunction. Our hypothesis is that dietary lipids negatively regulate SIRT1 by activating an innate immune response via the endotoxin/TLR4 axis. To test this hypothesis, mice were fed a single dose of polyunsaturated fat (corn oil) or monounsaturated fat (olive oil). Subsequently, mice were subjected to intravital microscopy to quantify vascular dysfunction in intestinal postcapillary venules and arterioles in WT mice, mice genetically deficient in TLR4 (TLR4^-/-^) and mice pre-treated with antibiotics to sterilize the gut of gram negative bacteria and endotoxin. Our results demonstrate a potential link between the TLR4/endotoxin axis and expression of SIRT1 during the postprandial phase. These findings provide new insights into the genesis of vascular stress and the potential involvement of epigenetic controls leading to the induction of a ‘stressed’ microvascular phenotype.

## Methods

### Ethics statements

All procedures using animals were reviewed and approved by the Institutional Animal Care and Use Committee (IACUC) of LSU Health Sciences Center and were performed according to the criteria outlined by National Institutes of Health guidelines. All surgery was performed under ketamine and xylazine anesthesia, and all efforts were made to minimize suffering.

### Animals

Wild-type (WT) C57/BL6J and B6.B_10_ ScN-Tlr4^lps-del^/JthJ (TLR4^-/-^) mice, weighing 17-22 g, the ages of 6-8 weeks, were obtained from the Jackson Laboratory (Bar Harbor, ME). The mice were housed in a temperature- and humidity-controlled room and were allowed free access to a standard chow diet and water before the experiments.

### Experimental groups

The following 7 experimental groups were included in this study: **1)** saline-treated WT mice (n = 10); **2)** WT mice + 10 ml/kg olive oil (n = 12); **3)** WT mice + antibiotics (pre-treated with 450 mg/kg/day polymyxin B and 150 mg/kg/day neomycin by gavage for 7 days) + 10 ml/kg olive oil (n = 3); **4)** TLR4^-/-^ mice + 10 ml/kg olive oil (n = 3); **5)** WT mice + 10 ml/kg corn oil (n = 12) **6)**. WT mice + antibiotics (pre-treated with 450 mg/kg/day polymyxin B and 150 mg/kg/day neomycin by gavage for 7 days) + 10 ml/kg corn oil; (n = 3) **7)** TLR4^-/-^ mice + 10 ml/kg corn oil (n = 3). Intravital microscopy was performed 1.0, 1.5, 2.0, 2.5 hours after oil administration in 2) and 5) groups. In 3) and 4) groups, intravital microscopy was performed only at 2.0 h. In 6) and 7) groups, intravital microscopy was performed just at 1.5 h.

### Platelet preparation

Approximately 0.9 ml of blood was collected from corresponding donor mice through a carotid artery cannula (polyethylene tubing, PE-10) into a polypropylene tube containing 0.1 ml of acid-dextrose buffer (Sigma; St. Louis, MO). The tube was centrifuged at 1,200 rpm for 8 min. The supernatant was transferred into another polypropylene tube and centrifuged again at 1,200 rpm for 3 min to remove any residual RBCs. This supernatant was transferred to another new tube and centrifuged at 3,000 rpm for 10 min to separate the platelets from the plasma. The platelets were suspended in PBS, counted, and divided into aliquots that contained 1-1.5 million platelets. Before injection into recipient mice, the platelets were incubated for 10 min with the fluorochrome CFSE, Molecular Probes, Eugene, OR) at a final concentration of 90 μM. The platelet suspension was then centrifuged at 3,000 rpm for 10 min, resuspended in 250 μl PBS and stored in the dark until injected into mice [20].

### Surgical procedure

The procedures used to evaluate blood cell adhesion and rolling in the murine intestinal microvasculature have been described previously [20]. Briefly, the animals were anesthetized subcutaneously using a mixture of ketamine and xylazine at a dose of 100 and 5 mg/kg, respectively. The right jugular vein was cannulated with polyethylene tubing (PE-10) for the administration of platelets. Core body temperature was maintained at 35 ± 0.5°C using an external temperature controlled heat lamp.

### Intravital microscopy

Following a midline incision, a small portion of the jejunum along with attached mesentery was carefully exteriorized, placed across a viewing cover glass, and superfused on a thin sheet of glass through which the bowel wall could be visualized. After a 20-min stabilization period, the CFSE-labeled platelets were injected via the jugular vein. 5 minutes later, rhodamine-6G (0.02%) was administered via the jugular vein for fluorescent labeling and visualization of leukocytes.

Fluorescently labeled platelets and leukocytes were visualized with an OLYMPUS IX71 inverted microscope equipped with a 75-W XBO xenon lamp and using a 20× objective. Visualization was accomplished using a filter with an excitation of 470-490 nm, a dichroic mirror (510 nm) with images recorded using a SONY DXC-390 video camera for offline evaluation. A 1-min recording of a 200-um length of three to five jejunal venules were obtained from each mouse. The total number of rolling and adherent leukocytes was determined, as well as the number of rolling and adherent platelets. Leukocytes and platelets were counted as ‘rolling’ if they were moving at a velocity significantly slower than the centerline velocity of the microvessel. ‘Adherent’ leukocytes or platelets were defined as cells remaining stationary on the vessel wall for ≥30 seconds and expressed as the number of cells per second per square millimeter of venular surface, calculated from diameter and length, and assuming cylindrical vessel shape. Venular diameter was measured with a video caliper.

Once the venular data were collected, the animals were allowed to stabilize for 20-30 min, and the arterioles with diameters between 15-40 μm and a wall shear rate (WSR) of ≥500/s were chosen for study. The diameters and red blood cell velocities were measured in the chosen sections before and after superfusion with 10^-5^ M of the endothelium-dependent vasodilator Ach for 5 min. The arterioles were then superfused with BBS and allowed to return to baseline values. Arterioles were then exposed to 10 μM papaverine (an endothelial-independent vasodilator) to determine maximal dilation. Arteriolar vasorelaxation responses to Ach were expressed as the % change in diameter normalized to the baseline.

### Western blotting assay

Total protein (100 μg) was extracted from frozen samples, separated on 10% SDS/PAGE gels and transferred to PVDF membranes. Membranes were blotted with anti-TLR4 (Cell Signaling Technology; Danvers, MA), anti-SIRT1 (Santa Cruz Biotechnology), anti**-**NF-κB p65 (Cell Signaling Technology; Danvers, MA), anti-pNF-κB p65 (Cell Signaling Technology; Danvers, MA) and β-actin (AbD-Serotec) overnight. Target proteins were visualized using ECL-Plus detection reagents (Amersham Biosciences; Piscataway, NJ) in a Chemidox XRS documentation system (Bio-Rad Laboratories; Hercules, CA).

### Statistical analysis

Statistical analyses of the data were performed with using one-way ANOVA with Fisher’s post hoc test. All values are reported as means ± SEM. Statistical significance was set at p < 0.05.

## Results

### The effect of dietary oils on leukocyte/platelet trafficking during postprandial lipidemia

**Figure 1:**
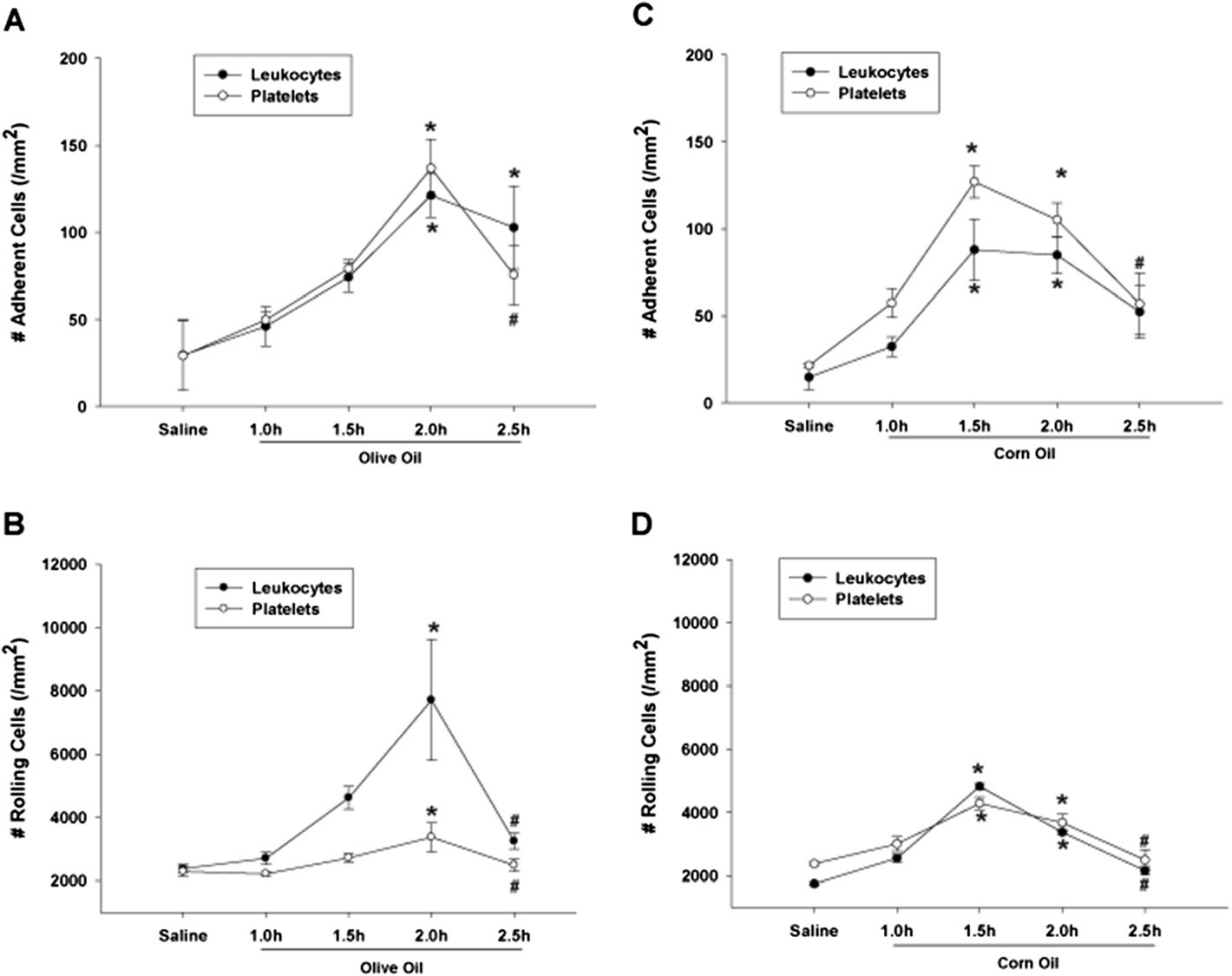
**Time dependent effects of feeding dietary oils on leukocyte and platelet adhesion and rolling in jejunum venules of WT C57/BL6J mice.** The extent of leukocyte and platelet trafficking in post-capillary venules in the jejunum wall of WT C57/BL6J mice was determined at various time points (1.0 h, 1.5 h, 2.0 h and 2.5 h) following gavage with olive oil or corn oil by intravital microscopy. **[A]** Leukocyte and platelet adherence to post-capillary venules was significantly increased 2 h after olive oil administration (*P < 0.05 vs. Saline control). Platelet adherence significantly declined by 2.5 h post following treatment (^#^P < 0.05 vs. Olive 2.0 h group) and leukocyte adherence began to decline at 2.5 h but it was still higher than saline control (*P < 0.05 vs. Saline control) **[B]** Leukocyte and platelet rolling in post-capillary venules was also significantly increased 2.0 h after gavage with olive oil (*P < 0.05 vs. Saline control) and then significantly declined after 2.5 h. (^#^P < 0.05 vs. Olive 2.0 h group) **[C]** Leukocyte and platelet adherence to post-capillary venules was significantly increased at 1.5 h, 2.0 h and 1.5 h was the peak time point for corn oil (*P < 0.05 vs. Saline control). They began to decline from 2.0 h post following gavage and platelet adherence significantly declined by 2.5 h post following treatment (^#^P < 0.05 vs. Corn oil 1.5 h group) **[D]** Leukocyte and platelet rolling in post-capillary venules was also significantly increased at 1.5 h and 2.0 h (*P < 0.05 vs. Saline control) and then significantly declined after 2.5 h. (^#^P < 0.05 vs. Corn 1.5 h group).

The extent of leukocyte and platelet trafficking in post-capillary venules in the jejunum wall of WT C57/BL6J mice was determined at various time points (1.0 h, 1.5 h, 2.0 h and 2.5 h) following gavage with olive oil or corn oil by intravital microscopy. Leukocyte and platelet adherence to post-capillary venules was significantly increased 2 h after olive oil administration. Platelet adherence significantly declined by 2.5 h post following treatment and leukocyte adherence began to decline at 2.5 h but it was still higher than saline control (Figure 1A). Leukocyte and platelet rolling in post-capillary venules was also significantly increased 2.0 h after gavage with olive oil and then significantly declined after 2.5 h (Figure 1B). Leukocyte and platelet adherence to post-capillary venules was significantly increased at 1.5 h, 2.0 h and 1.5 h was the peak time point for corn oil. They began to decline from 2.0 h post following gavage and platelet adherence significantly declined by 2.5 h post following treatment (Figure 1C). Leukocyte and platelet rolling in post-capillary venules was also significantly increased at 1.5 h and 2.0 h and then significantly declined after 2.5 h (Figure 1D). These results suggested feeding mono-unsaturated olive oil or polyunsaturated corn oil promotes leukocyte and platelet trafficking in the microvasculature and only difference is peak time point.

### Endotoxin plays a causal role in vascular dysfunction during postprandial lipidemia

**Figure 2:**
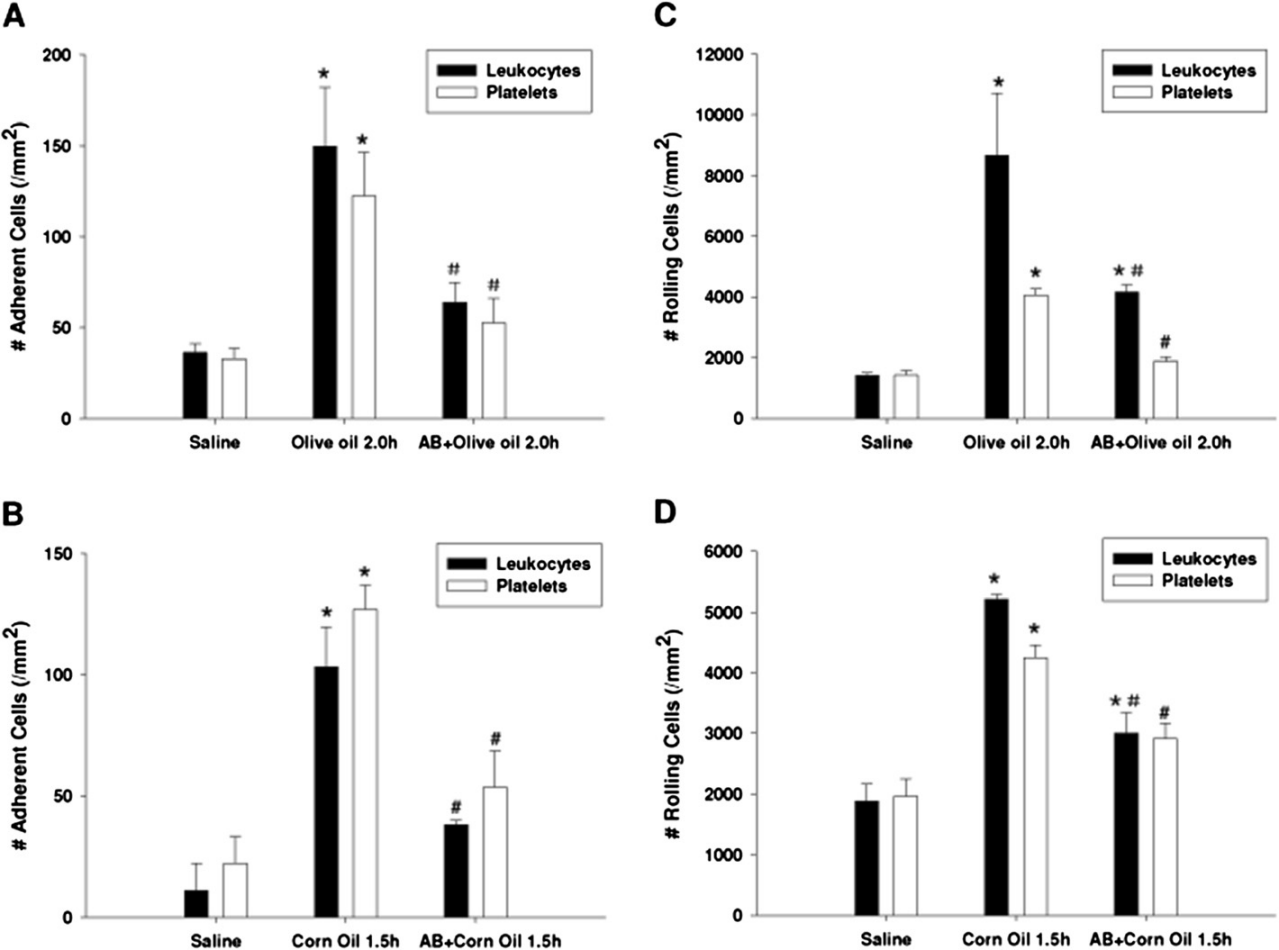
**Antibiotics diminished leukocyte and platelet adherence and rolling induced by feeding dietary oils in jejunum venules of WT C57/BL6J mice.** Mice were pre-treated with saline or antibiotics (450 mg/kg/day polymyxin B and 150 mg/kg/day neomycin) by gavage for 7 days. After 7 days, olive oil or corn oil was administered and mice subjected to intravital microscopy at ‘peak’ time point respectively (2.0 h after olive oil gavage or 1.5 h after corn oil gavage). Antibiotics significantly diminished leukocyte and platelet adherence to post-capillary venules 2 h following gavage with olive oil (*P < 0.05 vs. Saline control. ^#^P < 0.05 vs. Olive 2.0 h group) **[A]** or 1.5 h after gavage with corn oil (*P < 0.05 vs. Saline control. ^#^P < 0.05 vs. Corn 1.5 h group) **[B]**. Antibiotics also significantly reduced leukocyte and platelet rolling in post-capillary venules induced by gavage with olive oil for 2 h (*P < 0.05 vs. Saline control. ^#^P < 0.05 vs. Olive 2.0 h group) **[C]** or with corn oil for 1.5 h (*P < 0.05 vs. Saline control. ^#^P < 0.05 vs. Corn 1.5 h group) **[D]**.

Endotoxin has long been known to contribute to the induction of several inflammatory states. Our previous study showed the antibiotics polymyxin B and neomycin can successfully blunt alcoholic hepatitis in rodents. Therefore, we wished to evaluate whether gut sterilization using these agents could also reduce vascular stress defects associated with postprandial lipidemia. Mice were pre-treated with saline or antibiotics (450 mg/kg/day polymyxin B and 150 mg/kg/day neomycin) by gavage for 7 days to reduce the TLR4 ligand endotoxin and also eliminate gut-derived gram negative bacteria, the source of this endotoxin. After 7 days, oil was administered and mice subjected to intravital microscopy. Our study shows that antibiotics significantly diminished leukocyte and platelet adherence to post-capillary venules 2 h following gavage with olive oil (Figure 2A) or 1.5 h after gavage with corn oil (Figure 2B). Antibiotics also significantly reduced leukocyte and platelet rolling in post-capillary venules induced by gavage with olive oil for 2 h (Figure 2C) or with corn oil for 1.5 h (Figure 2D).

### The role of TLR4 signaling during postprandial lipidemia

The TLR family of pattern recognition receptors is critical in host defense against invading pathogens. Ligand interactions with the TLR4 complex result in the recruitment of multiple adaptor molecules to the cell membrane that propagate signaling which result in the translocation of NF-kB to the nucleus leading to production of several inflammatory mediators. Since endotoxin is an important TLR4 ligand, we further hypothesized those vascular defects seen during postprandial lipidemia required TLR4 activation.

**Figure 3:**
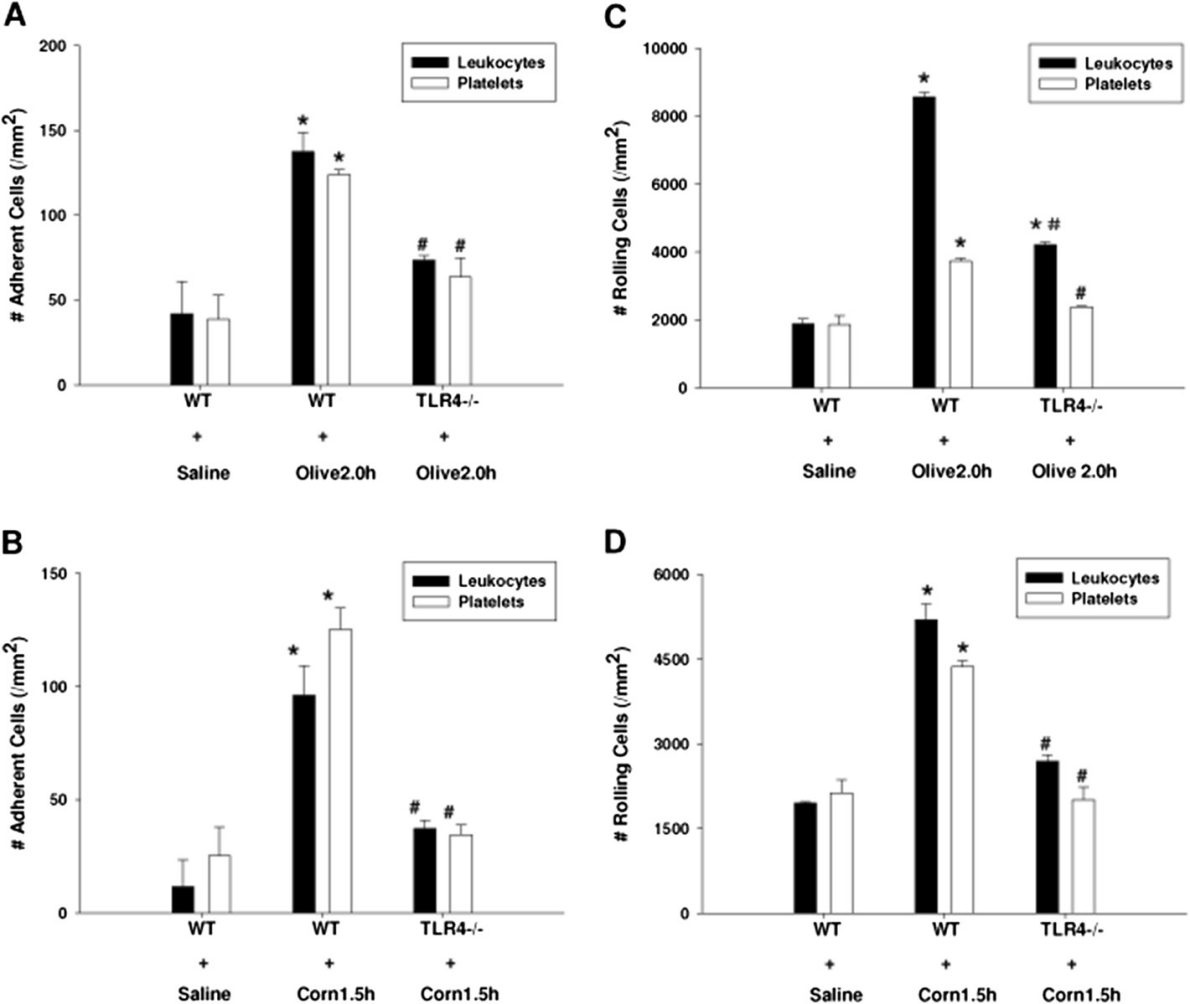
**TLR4 deletion diminished leukocyte and platelet adherence and rolling induced by feeding dietary oils in jejunum venules of TLR4**^**-/- **^**mice.** B6.B_10_ ScN-Tlr4^laps-del^/JthJ (TLR4^-/-^) mice were used as a TLR4-knockout strain, with wild type mice of this strain serving as controls. Intravital microscopy analysis of the jejunum venules was studied in these mice at their ‘peak’ time point respectively (2.0 h after olive oil gavage or 1.5 h after corn oil gavage). TLR4 deletion significantly diminished leukocyte and platelet adherence to post-capillary venules 2 h after gavage with olive oil (*P < 0.05 vs. WT + Saline control. ^#^P < 0.05 vs. WT + Olive 2.0 h group) **[A]** or 1.5 h after gavage with corn oil (*P < 0.05 vs. WT + Saline control. ^#^P < 0.05 vs. WT + Corn 1.5 h group) **[B]**. TLR4 deletion also significantly reduced leukocyte and platelet rolling in post-capillary venules induced by gavage with olive oil for 2 h (*P < 0.05 vs. WT + Saline control. ^#^P < 0.05 vs. WT + Olive 2.0 h group) **[C]** or with corn oil for 1.5 h (*P < 0.05 vs. WT + Saline control. ^#^P < 0.05 vs. WT + Corn 1.5 h group) **[D]**.

To investigate this, we used B6.B_10_ ScN-Tlr4^lps-del^/JthJ (TLR4^-/-^) mice as a TLR4-knockout mouse model, with wild type mice of this strain serving as controls. Intravital analysis of the gut microvasculature was studied in these mice at their ‘peak’ time points respectively. Our study showed that TLR4 deletion significantly diminished leukocyte and platelet adherence to post-capillary venules 2 h after gavage with olive oil (Figure 3A) or 1.5 h after gavage with corn oil (Figure 3B). And Leukocyte and platelet rolling again exhibited a pattern of response which was similar to that of leukocyte and platelet adherence (Figure 3C and 3D).

**Figure 4:**
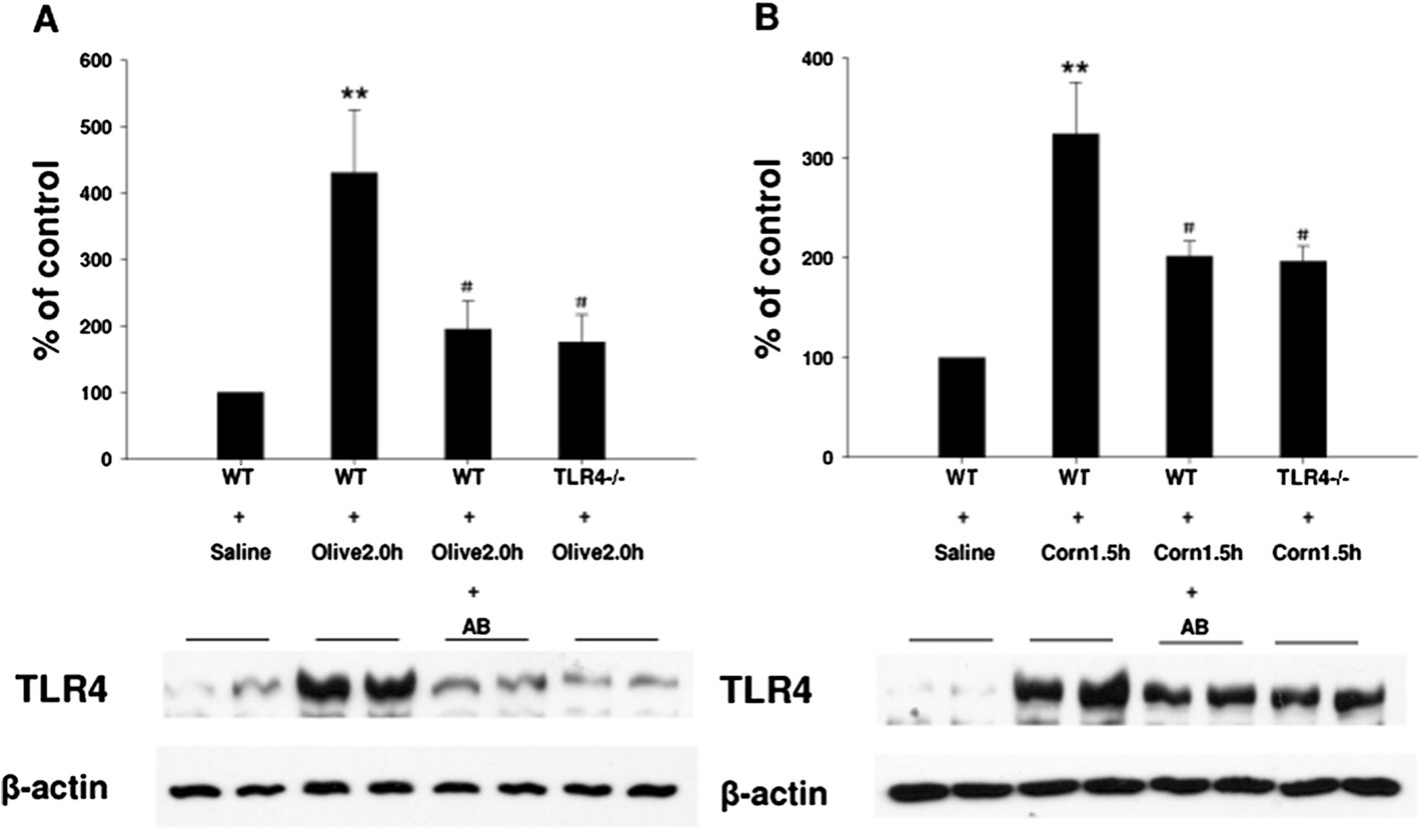
**Effect of feeding dietary oils on TLR4 protein expression during postprandial lipemia.** Samples of jejunum were collected following 2.0 h olive oil administration in WT + Saline, WT + Olive 2.0 h, WT + Olive 2.0 h + AB (pretreated with antibiotics for 7d) and TLR4-/- + Olive 2.0 h mice. TLR4 protein level was examined using standard western blotting techniques. TLR4 protein expression was significantly increased in mice gavaged with olive oil at 2 h (**P < 0.01 vs. WT + Saline control); this effect was significantly reduced in mice pretreated with antibiotics and in TLR4 knockout (TLR4^-/-^) mice (^#^P < 0.05 vs. WT + Olive 2.0 h group) **[A]**. Samples of jejunum were collected following 1.5 h corn oil administration in WT + Saline, WT + Corn 1.5 h, WT + Corn 1.5 h + AB (pretreated with antibiotics for 7d) and TLR4-/- + Corn 1.5 h mice. TLR4 protein level was examined using standard western blotting techniques. TLR4 protein expression was significantly increased in mice gavaged with corn oil at 1.5 h (**P < 0.01 vs. WT + Saline control); this effect was significantly reduced in mice pretreated with antibiotics and in TLR4 knockout (TLR4^-/-^) mice (^#^P < 0.05 vs. WT + Corn 1.5 h group) **[B]**.

**Figure 5:**
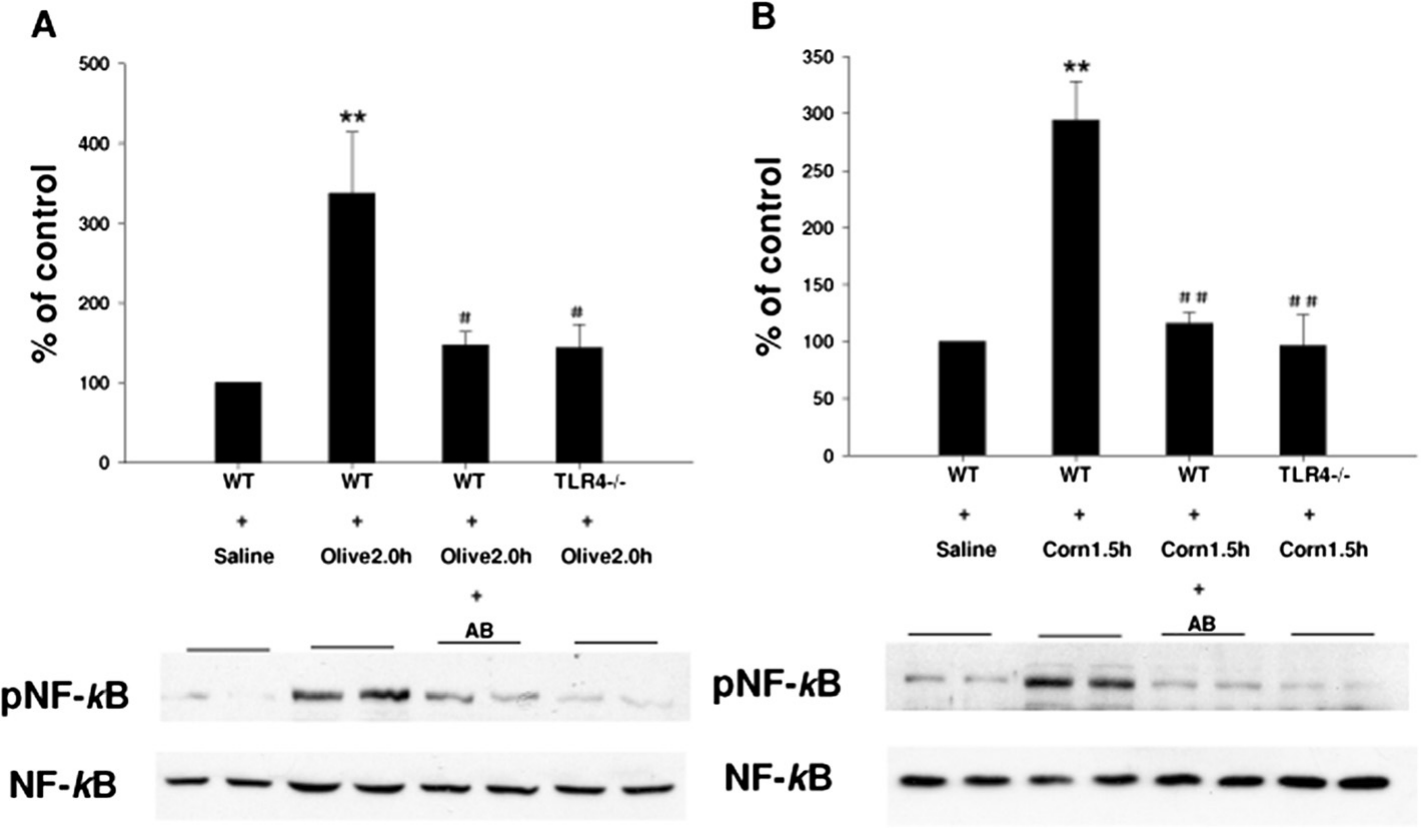
**Effect of feeding dietary oils on NF-*k*B p65 protein expression and phosphorylation during postprandial lipemia.** Samples of jejunum were collected following 2.0 h olive oil administration in WT + Saline, WT + Olive 2.0 h, WT + Olive 2.0 h + AB (pretreated with antibiotics for 7d) and TLR4-/- + Olive 2.0 h mice. NF-*k*B p65 protein expression and phosphorylation was examined using standard western blotting techniques. pNF-κB p65 expression was significantly increased in mice gavaged with olive oil at 2 h (**P < 0.01 vs. WT + Saline control); this phosphorylation was significantly decreased in mice which had been pre-treated with antibiotics and in TLR4 knockout (TLR4^-/-^) mice (^#^P < 0.05 vs. WT + Olive 2.0 h group) **[A]**. Samples of jejunum were collected following 1.5 h corn oil administration in WT + Saline, WT + Corn1.5 h, WT + Corn 1.5 h + AB (pretreated with antibiotics for 7d) and TLR4-/- + Corn 1.5 h mice. NF-*k*B p65 protein expression and phosphorylation was examined using standard western blotting techniques. NF-*k*B p65 expression was significantly increased in mice gavaged with corn oil at 1.5 h (**P < 0.01 vs. WT + Saline control); this effect was significantly reduced in mice pretreated with antibiotics and in TLR4 knockout (TLR4^-/-^) mice (^#^P < 0.05 vs. WT + Corn 1.5 h group) **[B]**. However, total NF-*k*B p65 protein expression was not significantly different between these groups.

We next explored the influence of feeding dietary oils on TLR4 and NF-kB protein expression and phosphorylation status. Samples of jejunum were collected following 1.5 h (for corn oil) or 2.0 h (for olive oil) administration. TLR4 and NF-kB protein levels and phosphorylation were examined using standard western blotting techniques. As shown in Figure 4A TLR4 protein expression was significantly increased in mice gavaged with olive oil at 2 h; this effect was significantly reduced in mice gavaged for 7 days with antibiotics (to clear endotoxin) and in TLR4 knockout (TLR4^-/-^) mice. Corn oil treated mice exhibited a pattern of response similar to olive oil (Figure 4B). Our studies also showed that NF-κB p65 phosphorylation (pNF-κB p65) was significantly increased in mice gavaged with olive oil for 2 h. This phosphorylation was significantly decreased in mice which had been pre-treated with antibiotics and in TLR4 knockout (TLR4^-/-^) mice. However, total NF-kB p65 protein expression was not significantly different between these groups (Figure 5A). Corn oil showed a similar pattern of response only 1.5 h after gavage (Figure 5B).

### Effects of dietary oils, endotoxin and TLR4 signaling on endothelium-dependent arteriolar vasodilatation responses during postprandial lipidemia

**Figure 6:**
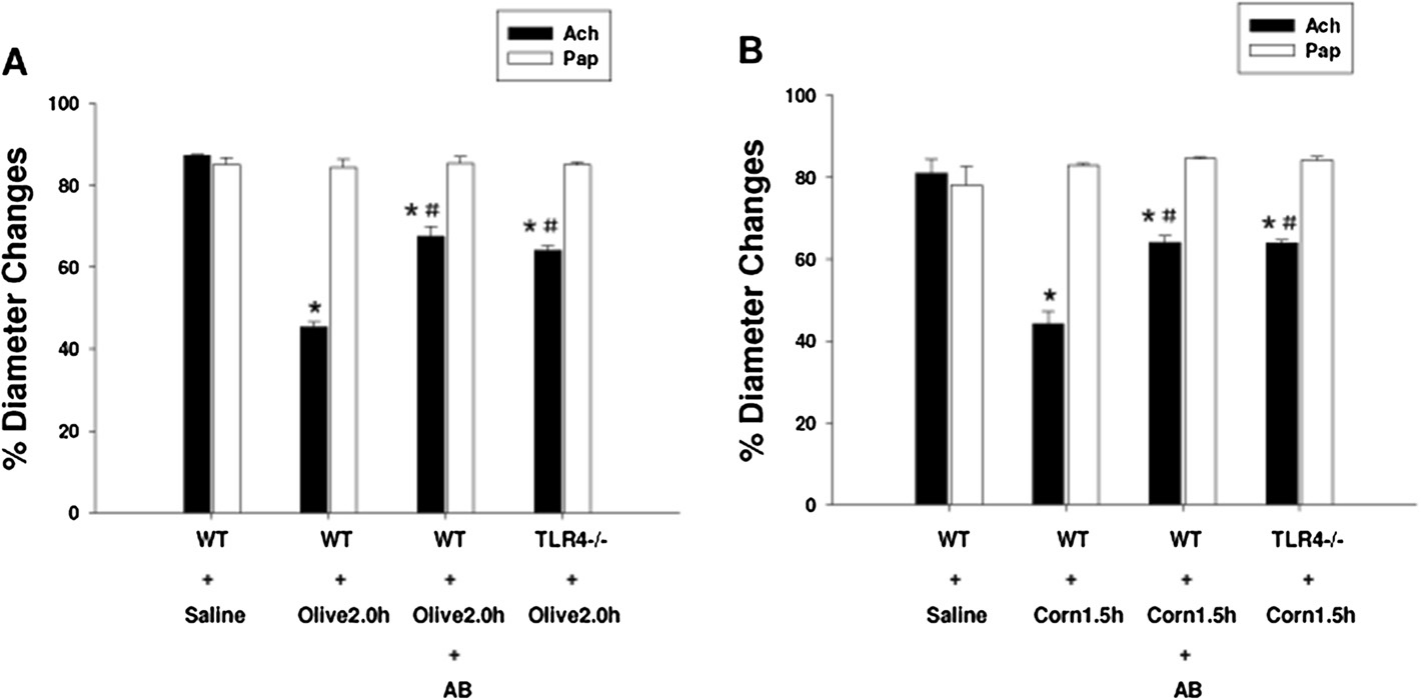
**Arteriolar vasodilatation responses to Ach (10**^**-5 **^**M) and Pap in different oil groups. [A]** The ability of arterioles to dilate in response to acetylcholine (Ach) and Pap in WT + Saline, WT + Olive 2.0 h, WT + AB + Olive 2.0 h and TLR4^-/-^ + Olive 2.0 h mice was measured by intravital microscopy. Arterioles of mice gavaged with olive oil for 2 h exhibited significantly impaired vasodilatation responses to Ach (*P < 0.05 vs. WT + Saline control). This phenomenon was however blunted in mice pre-treated with antibiotics and in TLR4 mutant mice but vasodilatory responses to papaverine, an endothelial-independent vasodilator, was not significantly different between these groups. (^#^P < 0.05 vs. WT + Olive 2.0 h group). **[B]** The ability of arterioles to dilate in response to acetylcholine (Ach) and Pap in WT + Saline, WT + Corn 1.5 h, WT + AB + Corn 1.5 h and TLR4^-/-^ + Corn 1.5 h mice was measured by intravital microscopy. Arterioles of mice gavaged with corn oil for 1.5 h exhibited significantly impaired vasodilatation responses to Ach (*P < 0.05 vs. WT + Saline control). This phenomenon was however blunted in mice pre-treated with antibiotics and in TLR4 mutant mice but vasodilatory responses to papaverine, an endothelial-independent vasodilator were not significantly different between these groups. (^#^P < 0.05 vs. WT + Corn 1.5 h group).

Impaired vasomotor activity is an early indicator of vascular dysfunction which can be demonstrated as an inability of the arteries and arterioles to vasodilate in response to endothelial dependent vasodilators. We therefore measured the ability of arterioles to dilate in response to Ach in each of the oil groups to investigate vascular dysfunction produced in response to these treatments. Our study shows that arterioles of mice gavaged with olive oil for 2 h exhibited significantly impaired vasodilation responses to Ach (compared to mice gavaged with saline). This phenomenon was however blunted in mice pre-treated with antibiotics and in TLR4 mutant mice (Figure 6A). Again similar responses were found in corn oil treated mice (Figure 6B). Our studies also showed that vasodilatory responses to papaverine, an endothelial-independent vasodilator were not significantly different between these groups.

### The effect of dietary oils, endotoxin and TLR4 signaling on SIRT1 expression during postprandial lipidemia

**Figure 7:**
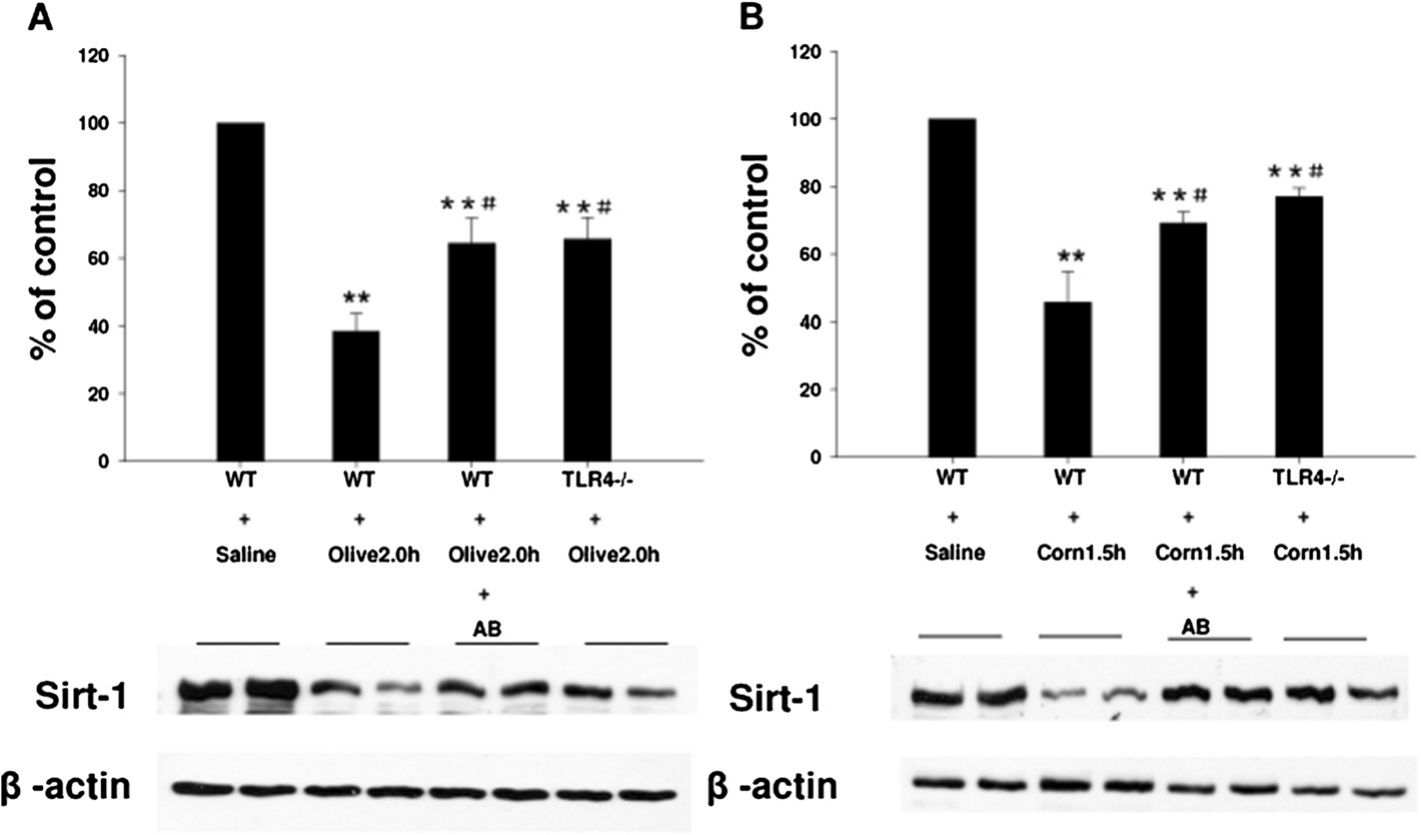
**Effect of feeding dietary oils on SIRT1 protein expression during postprandial lipemia.** Samples of jejunum were collected following 1.5 h (for corn oil) or 2.0 h (for olive oil) administration in each of the oil groups. SIRT1 protein expression was examined using standard western blotting techniques. **[A]** SIRT1 protein expression is significantly diminished by feeding olive oil for 2 h (**P < 0.01 vs. WT + Saline control), a phenomenon that is attenuated in mice pre-treated with antibiotics and in TLR4^-/-^ mice (^#^P < 0.05 vs. WT + Olive 2.0 h group). **[B]** Corn oil treated mice exhibited similar pattern of response but at different peak time points (1.5 h after gavage). (**P < 0.01 vs. WT + Saline control. ^#^P < 0.05 vs. WT + Corn 1.5 h group).

We have recently shown that SIRT1 heterozygosis promoted fat accumulation and inflammation in mice which were fed a high fat diet [21]. However, the response of SIRT1 to fat quality and the potential role of TLR4 in SIRT1 regulation have not yet been investigated. Samples of jejunum were therefore collected after 1.5 h (following corn oil) or 2.0 h (following olive oil). SIRT1 protein levels examined using western blotting show that SIRT1 protein expression is diminished by feeding olive oil for 2 h, a phenomenon that is attenuated in mice pre-treated with antibiotics and in TLR4^-/-^ mice (Figure 7A). Corn oil treated mice exhibited similar pattern of response but at different peak time point (1.5 h after gavage) (Figure 7B).

## Discussion

Food intake is an important event that dramatically affects vascular reactivity. Short-term feeding trials have shown the potential of different foods to improve endothelial function, either as isolated nutrients, such as n-3 PUFA, L-arginine, and antioxidant vitamins, or as ‘healthy’ food patterns [12]. Conversely, high-fat meals are usually followed by transient endothelial dysfunction in association with elevated triglyceride-rich lipoproteins [13]. Impaired endothelial function is central to the atherosclerotic disease process, and represents a strong, independent risk factor for future cardiovascular disease and mortality [1,2]. The ingestion of a HFM acutely changes the blood lipid profile and reduces endothelial function for several hours after meal [3]. Because a significant proportion any lifetime is spent in the ‘postprandial’ state, postprandial signals which impair endothelial function may play a significant role in atherosclerotic disease progression.

Olive oil, a staple of the Mediterranean diet, has been presumed to have vaso-protective properties. However, this assumption is under dispute with some studies suggesting that olive oil may actually cause postprandial impairment of endothelial function leading to atherosclerosis and vascular stress [22,23]. Such endothelial dysfunction could reflect increased postprandial oxidative stress, as administration of antioxidant vitamins C and E together with olive oil appears to reverse this type of endothelial dysfunction [23]. Larsen et al. have reported that in a clinical study, olive oil activated coagulation factor VII to the same extent as butter [24]. Therefore, olive oil does not have a clearly beneficial, direct effect on vascular function. Robert et al., found that omega-9 (oleic acid)-rich olive oil significantly impairs endothelial function during the postprandial state [25]. Unfortunately, data about the postprandial effects of this diet on endothelial function, an early indicator of vessel damage and cardiovascular disease, are still limited. Therefore, we investigated possible postprandial effects of unsaturated fats on vascular function.

In this study we examined the efficacy of two types of unsaturated fat - mono-unsaturated olive oil and polyunsaturated corn oil on vascular dysfunction during postprandial lipidemia. Our current data confirm that feeding mono-unsaturated olive oil or polyunsaturated corn oil promoted leukocyte and platelet trafficking in the gut microvasculature, and impaired endothelium-dependent arteriolar vasodilator responses during postprandial lipidemia. Currently the molecular mechanisms underlying impaired vascular function in the postprandial state remain incompletely understood.

Our study further showed that abnormal vasoreactivity after fat meals is attenuated by antibiotic pretreatment (450 mg/kg/day polymyxin B plus 150 mg/kg/day neomycin, 7 days). This finding suggests that endotoxin (produced by gut microflora) plays a direct role in vascular dysfunction associated with postprandial lipidemia. Endotoxin is known to mediate several inflammatory and stress responses in the pathogenesis of several infectious and inflammatory states. Our previous study showed the antibiotics polymyxin B plus neomycin can successfully attenuate alcoholic hepatitis in rodents. Therefore, in this study, antibiotics were again administered to eliminate endotoxin as a TLR4 ligand, and to clear gut-derived gram negative bacteria, which are a source of endotoxin. This study showed that antibiotics successfully blocked development of vascular dysfunction produced by postprandial lipidemia. In other words, we suggest that endotoxin plays a direct causal role in vascular dysfunction mediated by postprandial lipidemia.

Since endotoxin is an important ligand for TLR4, we anticipated that vascular defects due to postprandial lipidemia would therefore require TLR4. Our study also showed that TLR4 gene-deletion significantly diminished leukocyte and platelet adherence and rolling on postcapillary venules following olive oil or corn oil administration. The reductions in endothelial vasodilatory responses to Ach were also blocked in TLR4 mutant mice. Furthermore, TLR4 protein expression was significantly increased in mice given olive or corn oil. This induction of TLR4 was significantly decreased in mice pre-treated with antibiotics and in TLR4 knockout (TLR4^-/-^) mice. Therefore TLR4 deletion conferred similar protection as antibiotics, consistent with TLR4-endotoxin signals as important mediators of postprandial lipid induced vascular stress.

The TLR family of pattern recognition receptors is critical in host defense against invading pathogens. Ligand interactions with the TLR4 complex result in the recruitment of multiple adaptor molecules to the cell membrane which activate NF-κB leading to inflammatory mediator release. Dietary oil feeding significantly increased pNF-κB p65 expression (not absolute NF-kB p65 levels); this activation was significantly attenuated by antibiotics or TLR4 knockout (TLR4^-/-^) mice. TLR4 – endotoxin signaling clearly plays a central role in vascular dysfunction during postprandial lipidemia.

We have recently shown that SIRT1 heterozygosity promoted fat accumulation and inflammation induced by high fat feeding [21]. Because resveratrol consumption appears to reduce atherosclerosis [26,27], and activates SIRT1, SIRT1 may mediate at least part of this protective response [16]. Cell culture and rodent studies demonstrate that SIRT1: 1) protects against endothelial dysfunction by preventing stress-induced senescence [17]; 2) preserves eNOS dependent NO generation [18]; and 3) SIRT1 over expression suppresses high fat diet-induced endothelium-dependent dysfunction in apoE knockout mice [19]. While these data suggest that SIRT1 is cardio-protective, the mechanisms underlying these benefits are still not clear. Our study here shows that SIRT1 expression is diminished by olive or corn oil, which can also be blocked by antibiotics and by TLR4 gene-deletion. Taken together with our other findings, these studies are consistent with dietary oils as negative regulators of SIRT1 which activate the innate immune response through the endotoxin/TLR4 axis.

The present study provides important insights into molecular mechanisms underlying fatty acid-induced vascular dysfunction. We have demonstrated that epigenetic mechanisms mediated by SIRT1 are of pivotal importance in the development of vascular dysfunction and that dysregulation of these mechanisms occurs through endotoxin-elicited inflammatory signals via TLR4. This finding challenges the concept that olive oil has a vaso-protective effect and provides a unique prospective about the “beneficial effects” of the dietary oils.

## Conclusions

Our data suggest that dietary oils may be negative regulators of SIRT1 which activate the innate immune response through the endotoxin/TLR4 axis. These findings establish a link between innate immunity (i.e. the endotoxin/TLR4 axis) and epigenetic controls mediated by SIRT1 in the genesis of diet associated vascular stress. This provides an important new paradigm which may provide several bases of novel treatment modalities for cardiovascular disease.

## Competing interests

The authors declare that they have no competing interests.

## Authors’ contributions

CAR contributed to the study design and interpretation of the data. PY was responsible for the main experimental work, data collection, interpretation of the data and preparation of the manuscript. JSA critically revised and wrote the manuscript. All authors read and approved the final manuscript.
